# The Impact of Using mHealth Apps on Improving Public Health Satisfaction during the COVID-19 Pandemic: A Digital Content Value Chain Perspective

**DOI:** 10.3390/healthcare10030479

**Published:** 2022-03-04

**Authors:** Junwei Cao, Guihua Zhang, Dong Liu

**Affiliations:** 1 Department of Digital Convergence Business, Yeungnam University, Gyeongsan 38542, Korea; 21850211@yu.ac.kr; 2 Department of Business, Yeungnam University, Gyeongsan 38542, Korea; 3 Department of Global Business, Yeungnam University, Gyeongsan 38542, Korea; bruceliu@yu.ac.kr

**Keywords:** mobile health app, public health, digital content value chain, COVID-19

## Abstract

The use of mobile technology and equipment has been found to be successful in the governance of public health. In the context of the coronavirus disease 2019 (COVID-19) pandemic, mobile health (mhealth) apps are expected to play an important role in the governance of public health. This study establishes a structural equation model based on the digital content value chain framework, identifies the main values created by mhealth apps in the prevention and control of COVID-19, and surveys 500 citizens of China. The data were analyzed using an independent *t*-test and partial least squares structural equations (PLS-SEM). The results showed that people who use mhealth apps are more satisfied with public health governance than those who do not; the healthcare assurance value of mhealth apps and healthcare confidence positively influence the interaction between users and mhealth app functions, the interaction with information, and the interaction with doctors to improve users’ satisfaction with public health governance; and the parasocial relationships between doctors and users of mhealth apps positively affect the interactions between users and doctors to improve users’ satisfaction with public health governance. This study confirms the potential of mhealth apps toward improving public health governance during the COVID-19 pandemic from a new perspective and provides a new theoretical basis whereby mobile technology can contribute toward improving public health governance.

## 1. Introduction

Public health governance aims to prevent diseases, extend life, and promote health through organized social efforts, and it focuses on the impact of social conditions on health, such as the health system, social conditions, and the link between inequality and poor health [1]. The emergence of coronavirus disease 2019 (COVID-19) has compelled the world to invest a substantial amount of anti-epidemic materials, which has exacerbated the pressure on public health and medical resources. It even affects daily medical care [2]. Meanwhile, to reduce the risk of cross-infection, people are reluctant to visit medical institutions, even when they have health problems. This also complicates public health governance [3].

The use of mobile technology and mobile devices in public health governance has been proven successful [4,5]. Medical and public health services that are provided to the public through mobile phones, patient testing equipment, personal digital assistants, and other wireless devices are referred to as mobile health (mhealth) [6]. Currently, mhealth mainly provides services based on the form of a smartphone app [7,8]. The emergence of mhealth apps has changed the supply mode of health services and brought about benefits for both healthcare providers and recipients [9]. On the one hand, doctors use mhealth apps to process patient information and monitor patient health [10]. On the other hand, individuals use mhealth apps to obtain health information for immediate diagnosis [11].

Mobile health apps digitize traditional healthcare services and provide users with healthcare services via the Internet. The role of mhealth apps in public health management during the COVID-19 pandemic reflects the impact of their digital content (DC) value on society. Mobile health apps are expected to play an important role in the COVID-19 pandemic [12]. Therefore, many studies have indicated the important functional value of using mhealth apps for COVID-19 [2,3,12]. Presently, the COVID-19 epidemic recurs, thus overwhelming public health services, and thus, the pressure on public health governance remains at momentarily high levels [1], and the use of the mhealth apps allows patients to easily obtain health information and receive medical care, thus reducing the frequency of patient visits to the hospital and minimizing population mobility in areas of high risk [2,3,4]. Mobile health apps effectively promote information exchange, storage, and delivery, and they improve the ability of patients to monitor and respond to diseases [12,13,14,15]. They can also be used for training [16,17], information sharing [18,19,20], risk assessment [18], symptom self-management [16], contact tracking [18], family monitoring [21], and decision-making [3] during the COVID-19 pandemic.

Mhealth is a digital platform that deserves to be valued not only for its functional value but also for its emotional and social value [22]. In terms of emotional value, mHealth apps have proven to be an effective way to deliver mental health services [23,24,25,26]. A mobile medical app can significantly reduce stress and significantly improve people’s well-being by identifying emotional states and reducing loneliness [25]. In particular, the use of mHealth apps strengthens users’ resilience and makes it easier for them to get out of a difficult situation and maintain a positive mental state [26]. In terms of social value, the development of mHealth can alleviate the shortage of medical resources to a certain extent [27], improve the quality of medical services in remote areas and for vulnerable groups, and helps to maintain social harmony [28].

A review of the relevant literature revealed that (1) the current research has made many arguments for the functional value of the mhealth app in resisting COVID-19. However, there is a lack of research on its emotional and social values, especially for COVID-19, and the specific connotations manifested in these value dimensions of mhealth are even less clear. (2) The mechanisms of the impact of these values of the mhealth app on public health governance are also unclear. Therefore, the research questions of this study are presented as follows.

RQ1: What values do mhealth apps mainly create during the COVID-19 pandemic?

RQ2: How do the values created by mhealth apps improve public health governance during the COVID-19 pandemic?

To answer these questions, this study adopts the DC value chain framework combined with the relevant literature to construct a research model to analyze the value transfer of mhealth apps during the COVID-19 pandemic.

## 2. Theoretical Background and Hypothesis Development

### 2.1. Digital Content Value Chain Framework

With the rapid development of information technology, an increasing amount of traditional content is converted into DC for delivery [22]. Traditional content delivers physical value through physical value streams, while DC delivers digital value through DC value streams [29].

Digital content value streams refer to the entire DC process, from generation to use in a computer-mediated network. Digital content value streams encompass three stages: DC value creation, DC value interaction, and DC use [22,29,30]. Digital content promotes interactions between users and DC by creating new values. Ultimately, users create new values through DC use [22].

**Figure 1:**
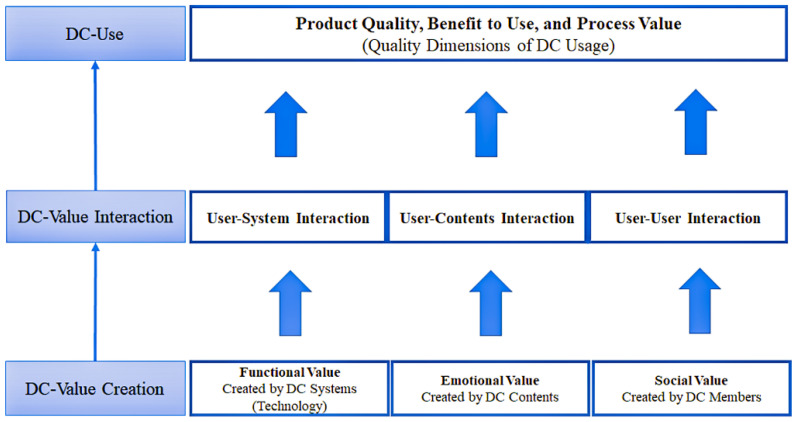
Digital content value chain framework.

To clarify the relationship between the various parts of the DC value stream, Kim and Kim [22] proposed a DC value chain framework based on the DC value stream. As shown in Figure 1, in the DC value creation stage, the DC system, DC, and DC users dominate, thereby creating the functional, emotional, and social DC values. In the DC value interaction stage, the user interacts with the system, content, and members because of the DC value and enters the user stage through the intermediary effect of interaction. Users create a new value based on the value given by the DC (such as performance improvement and satisfaction with the product). The DC value chain framework analyzes the DC platform from the user’s perspective, can better understand the value transfer process in a specific DC platform, and more intuitively analyzes the user’s perception of the DC value.

Mobile health apps digitize traditional healthcare services and provide users with healthcare services and medical knowledge through the Internet. They have developed from the earliest apps that can only provide a single service to DC-providing platforms for multiple services, such as appointment registration, online diagnosis, drug purchase, and health knowledge search. Mobile health apps can monitor the user’s physical data and provide users with medical advice and instant online medical services, thereby alleviating the pressure on medical resources. These apps can also relieve users’ anxiety about diseases by providing corresponding health management information [31,32], as well as functional, emotional, and social values. Users gain value and create new value through interactions with mhealth app system functions, health information interactions, and member interactions. Therefore, it is essential to analyze the value delivery of mhealth apps during the COVID-19 pandemic based on the DC value chain framework perspective.

### 2.2. Main Values of mHealth App during the COVID-19 Pandemic

The most important functional value created by mhealth apps during the COVID-19 pandemic is the provision of effective medical protection. Mobile health apps were rather unpopular before the emergence of COVID-19 [33]. However, with the increasing prevalence of COVID-19, the public health department has been encouraging patients to avoid using face-to-face medical services as much as possible to prevent cross-infection and effectively use limited public health resources [3,34]. Therefore, many countries have begun to use mhealth apps on a large scale to provide consultation, monitoring, and care services for patients [3]. Mobile health apps allow for the exchange of two-way data between patients and healthcare personnel to realize remote medical consultation, psychological consultation, health education, and obtain medical protection. It meets users’ utilitarian medical needs [9,35]. Satisfaction with utilitarian needs can positively affect user intentions [36,37]. Users must meet utilitarian medical needs through frequent interactions with mhealth apps; therefore, we propose the following hypotheses:

**H1a.**  *The healthcare assurance capabilities of mhealth apps have positively affected the interactions between users and the health functions of mhealth apps during the COVID-19 pandemic.*

**H1b.**  *The healthcare assurance capabilities of mhealth apps have positively affected the interactions between users and the health information of mhealth apps during the COVID-19 pandemic.*

**H1c.**  *The healthcare assurance capabilities of mhealth apps have positively affected the interactions between users and mhealth app doctors during the COVID-19 pandemic.*

The most important emotional value created by mhealth apps is confidence. It has been confirmed that the ability of mhealth apps to give users confidence is an important dimension in evaluating its quality, and it positively affects users’ satisfaction with mhealth apps and their continued use intentions during the COVID-19 pandemic [38,39]. The COVID-19 pandemic can cause psychological problems. In particular, there has been no specific medicine for the treatment of new coronary pneumonia, which is more likely to cause depression, anxiety, insomnia, and other negative emotions [40]. As a health information platform, mhealth apps can deliver positive health information and provide users with psychological intervention [41,42], for example, enlightening people about the pathogenesis of COVID-19 and elucidating the epidemic prevention dynamics of the government and related organizations to reduce users’ doubts and give users confidence.

Confidence has also been proven to be an important indicator for evaluating the quality of mhealth apps and user interactions [39], and it significantly predicts user behavior by triggering positive emotions [43]. During the COVID-19 pandemic, the information in mhealth apps can boost the user’s confidence, the user’s evaluation of the interactive quality of mhealth apps may be improved, and users tend to interact with mhealth apps more. Therefore, this study proposes the following hypotheses:

**H2a.**  *During the COVID-19 pandemic, the healthcare confidence-giving value of mhealth apps has positively affected the interactions between users and the health functions of mhealth apps.*

**H2b.**  *During the COVID-19 pandemic, the healthcare confidence-giving value of mhealth apps has positively affected the interactions between users and mhealth apps’ health information.*

**H2c.**  *During the COVID-19 pandemic, the healthcare confidence-giving value of mhealth apps has positively affected the interactions between users and mhealth app doctors.*

During the COVID-19 pandemic, the social value of mhealth apps has manifested at the level of a positive doctor–patient relationship [44]. In face-to-face diagnosis and treatment, patients usually feel pressure, because they are passive. In telemedicine, patients feel that they have the initiative, which reduces the pressure to visit a doctor [45]. Several studies have proven that medical services in an information network environment are more patient-centric, which allows patients and doctors to collaborate better and improve their mutual satisfaction [45].

The active doctor–patient relationship in the mhealth app platform is that of the society criterion. Parasocial relationships refer to the emotional bonds formed between the audience and the media characters. It is a one-way relationship. Parasocial relationships improve the user’s recognition of the media and increase the user’s participation [46,47]. In the mhealth app, doctors are the “media people” on the platform. The platform displays their personal information to the users. Users can choose their favorite doctors according to their preferences and establish one-way connections (user–function interaction and user–information interaction). After the diagnosis is complete, the doctor will be unable to actively communicate with the user. This process is completely dominated by the user. The user participates in the interaction with his or her own positive imagination of the selected doctor, which can easily form parasocial relationships [48]. Second, the health data monitoring function of the mhealth app can provide more accurate health information (user–function interaction and user–information interaction) when users communicate with doctors [49,50]. A study confirmed that 80% of doctors are satisfied when patients show digital health information [51]. Finally, accurate health information provided by patients can improve a doctor’s diagnosis and treatment performance [44]. Consequently, patients’ satisfaction with doctors improves, and they are more willing to use the various interactive mechanisms of the mhealth app platform (user–function interaction, user–information interaction, and user–doctor interaction). Therefore, this study proposes the following hypotheses:

**H3a.**  *During the COVID-19 pandemic, the parasocial relationship between doctors and patients in mhealth apps has positively affected the interactions between users and the health functions of mhealth apps.*

**H3b.**  *During the COVID-19 pandemic, the parasocial relationship between doctors and patients in mhealth apps has positively affected the interactions between users and the health information of mhealth apps.*

**H3c.**  *During the COVID-19 pandemic, the parasocial relationship between doctors and patients in mhealth apps has positively affected the interactions between users and mhealth app doctors.*

The interaction between mhealth apps and users transfers the value to the users’ satisfaction with public health governance. The information systems success theory points out that the use of information systems and satisfaction will interact, which will eventually affect individuals or organizations and generate net benefits [52,53]. The digital value chain framework also confirms that increasing the interaction between the system and the user, between the content and the user, and between the user and the user can play a key role in improving users’ satisfaction, process efficiency, product quality, and use efficiency [22,29,54].

During the COVID-19 pandemic, users have solved their health concerns by continuously using the health service function of mhealth apps and improved their confidence in health management, thereby enhancing the interactions between doctors and patients, as well as forming a good doctor–patient relationship. The value created by mhealth apps through interactions may alleviate the medical pressure of patients during the epidemic and improve patients’ satisfaction with public health governance. Therefore, this study proposes the following hypotheses:

**H4a.**  *During the COVID-19 pandemic, the interaction between users and the health functions of mhealth apps positively affected users’ satisfaction with public health governance.*

**H4b.**  *During the COVID-19 pandemic, the interaction between users and the health information of mhealth apps has positively affected users’ satisfaction with public health governance.*

**H4c.**  *During the COVID-19 pandemic, the interaction between users and mhealth app doctors has positively affected users’ satisfaction with public health governance.*

## 3. Research Model and Questionnaire Survey

### 3.1. Digital Content-Value Chain Framework

**Figure 2:**
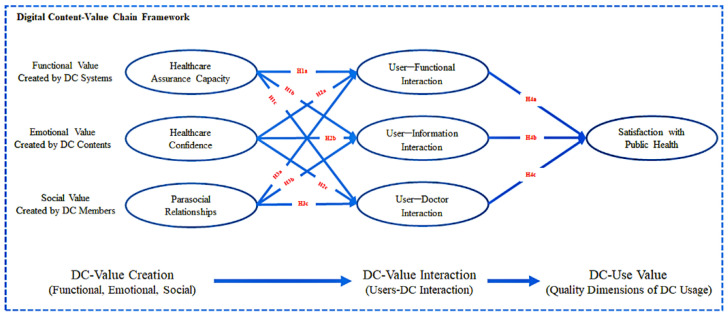
Proposed research model.

This study proposes a research model of the mhealth app value chain during the COVID-19 pandemic (Figure 2). The model shows that an mhealth app transfers the value created by itself to public health governance through interactions with users. Mobile health apps do not directly affect public health governance during the COVID-19 pandemic as an auxiliary medical mobile phone app, but they can provide users with functional (healthcare assurance), emotional (patient healthcare confidence), and social (patient–patient relationship) values that promote the interactions between users and mhealth apps, meet medical needs, increase confidence in health management, and establish a harmonious doctor–patient relationship, thereby increasing users’ satisfaction with the COVID-19 public health governance.

### 3.2. Questionnaire Survey

The questionnaire is designed according to the current research and the COVID-19 background. The questionnaire uses a 5-point Likert scale based on existing research and combined with the actual situations of the current research. All the questions are compulsory. If a question is unanswered, the questionnaire cannot be submitted. After the questionnaire is completed, we invite experts in the field of management information systems to investigate it, and 60 undergraduates are also invited to test it. The final questionnaire is presented in Appendix A.

Before we give out the questionnaires, we consult the school ethics committee to ensure that there are no ethical issues in the questionnaire. All the participants are informed of the following information: (1) The questionnaire is innominate. (2) The content and purpose of the questionnaire. (3) You have the right to answer or not answer. (4) No private information involved. (5) After completing the questionnaire, you will receive a gift.

A serious epidemic broke out in Yangzhou, China from 28 July to 30 August 2021. The epidemic prevention measures implemented by the government made people stay at home, which provided a good analysis environment for this study. This study employed an online questionnaire in Yangzhou from 20 August 2021 to 30 August 2021. By using the snowballing survey method, we randomly recruited 100 users who used mhealth apps during this period to conduct a survey and asked them to send the questionnaire to their friends. If the people who were investigated choose “mhealth app was not used in the epidemic”, they skipped the questions about mhealth and answered the questions about public health satisfaction. Finally, we received a total of 581 questionnaires after we eliminated 93 unqualified and invalid answers (e.g., answer time less than two minutes; more than 70% of the answers were the same), while the effective questionnaires are sorted out. Among them, 316 people used mhealth app health services, information search, online diagnosis, and other functions during this period and answered all the questions. A total of 172 people said they had not used mhealth apps during this period and only answered the questions on public health satisfaction.

**Figure 3:**
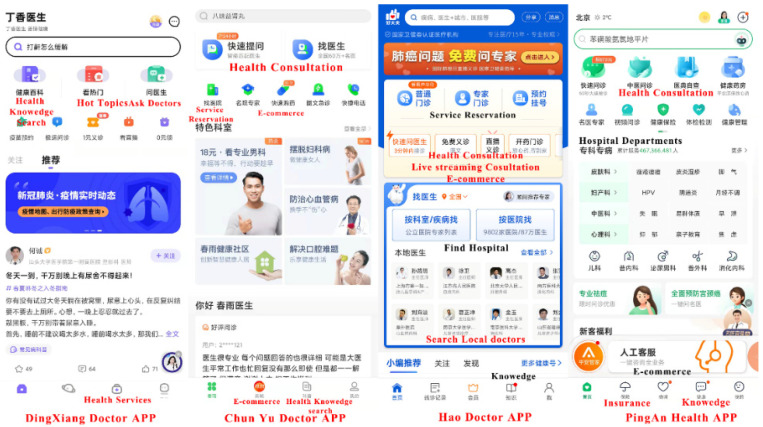
The main functions of respondents’ commonly used mhealth apps.

This study conducted a necessary demographic survey of people who have used mhealth apps (Table 1). Among the 316 people who had used these apps, 128 were male (40.5%) and 188 were female (59.5%). The proportion of people aged 31–40 was the highest (N = 87, 27.5%), followed by people aged 21–30 (N = 84, 26.6%). Among all the respondents, 140 (44.3%) had no higher education, 154 (48.7%) had a bachelor’s degree, and 22 (7%) had a master’s or doctoral degree. The monthly income of most respondents ranged from 2001 to 3000 yuan (USD 295–440) (N = 80, 25.3%), followed by those with a monthly income of 3001–4000 yuan (USD 441–558) (N = 71, 22.5%). In terms of app brands, 74 people used “Pingan Health”, 70 people used “Chunyu Doctor”, 66 people used “Dinxiang Doctor”, and remaining 106 people reported using other mhealth apps (e.g., Hao Doctors). These apps have some commonalities, as shown in Figure 3. They are all integrated apps with many functions, and one app can meet multiple healthcare needs. These functions include e-commerce, health knowledge search, service reservation, health consultation, task-specific processing, medical service review, etc.

To avoid a nonresponse bias, a paired samples *t*-test was conducted for the top 20 and bottom 20 respondents who submitted the questionnaire. The results showed no significant differences between the two groups.

## 4. Methods

There are two types of structural equation models (SEMs)—one is based on covariance (CB-SEM), and the other is based on variance (VB-SEM). In this study, the VB-SEM partial least squares SEM (PLS-SEM) and the corresponding software package (Smartpls3.0) were used. PLS-SEM is a second-generation multivariate data analysis method that is mainly used to carry out exploratory theoretical research. This method can ensure the integrity of all relationships between independent and dependent variables [55]. Compared with CB-SEM, (1) PLS-SEM is more suitable for models with more than six variables [56]; (2) PLS-SEM is good for processing small sample data [56]; and (3) PLS-SEM can process non-normally distributed data [56]. In summary, the PLS-SEM method is more suitable than the CB-SEM in the theoretical development stage, and it has been shown that PLS-SEM can replace the CB-SEM in most social science research cases [56] and is widely used in social, economic, and business research [57,58].

A multivariate normality analysis was performed on the data using a network calculator (http://www.biosoft.hacettepe.edu.tr/MVN/, accessed on 3 January 2022) [59]. The results of the multivariate normality analysis showed that Mardia’s multivariate skewness β = 75.184, *p* < 0.01 and multivariate kurtosis β = 788.253, *p* > 0.05, which suggest multivariate non-normality [60]. In addition, there are seven variables in this study. Therefore, it is suitable to use PLS-SEM for data analysis in this study.

## 5. Results

### 5.1. Pretest Results

This study measures the impact of the value created by mhealth apps on public health governance during the COVID-19 pandemic through the user’s satisfaction with public health governance after using mhealth apps. Therefore, it is necessary to survey people who have or have not used mhealth apps to determine whether there is a difference in the satisfaction of the population to public health governance. If there is no difference, it means that the value transfer created by mhealth apps does not exist, and there is no need for further analysis.

This study compares the satisfaction with public health governance of 316 people who have used mhealth apps and 172 people who have not through an independent sample *t*-test. The results are presented in Table 2. The average satisfaction of users who have used mhealth apps regarding public health governance during the COVID-19 pandemic is higher than that of those who have not used it, and the difference is significant.

### 5.2. Common Method Bias Test Results

Common method bias is a problem that can easily appear in the questionnaire. Harman’s single-factor analysis is widely used to detect common method deviations in social science research [61]. This method indicates that a single factor can be extracted. If the variance is less than 40%, it means that the survey data are less affected by the deviation of the commonly used methods [62]. The Harman data analysis conducted in this research shows that the ratio of extracted variables is 30.49% (less than 40%).

We further used the full variance inflation factor (VIF) test method to conduct a common method bias test on the data. Some studies have pointed out that all variables and dump variables in the model are subjected to the full VIF test. If the VIF value is greater than 3.3, the model may be affected by common method bias. If all the VIFs obtained by the full VIF test are equal to or lower than 3.3, it can be considered that the model has no common method bias [57,63]. This has been widely applied in various studies [60]. The full VIF test results of this study show that all the VIF values are less than 3.3. We consider the test results of the two common method bias methods. Therefore, the authors believe that common method bias is not a serious issue in this study.

### 5.3. Measurement Model Results

First, the composite reliability is used to evaluate internal consistency reliability. As Table 3 shows, the composite reliability (CR) value of each structure is greater than 0.7, and Cronbach’s α is also greater than 0.7, indicating that the questionnaire items have high reliability [64]. This study confirms convergent validity by evaluating the average variance extracted (AVE). When the AVE value is higher than 0.5, it is considered that the standard for convergence validity is met. In our model, the AVEs are all higher than 0.5, with the lowest value being 0.648, indicating that the scale has good convergence validity [64].

Second, this study tests the discriminating validity using the heterotrait–monotrait ratio (HTMT) test. The results are shown in Table 4, where the value between the variables meets the requirement of less than 0.85 [64]. This study continues testing discriminating validity through the Fornell–Larcker criterion test (comparing the square root of AVE with the correlation coefficient), and the square root of each variable is greater than the correlation coefficient with other variables, which meets the test requirements in this study (see Table 5). In addition, as shown in Table 3, the factor loading of all items in this study is higher than 0.7, with the lowest value being 0.750, which meets the requirements of the threshold standard [64]. These results indicate that the discriminative validity of the scale in this study meets these requirements [64].

Third, we tested the goodness of fit of the model. The fit degree is calculated by the square root of the product of the R^2^ mean of the communality, while the result of the goodness of fit must be higher than 0.1. If it is higher than 0.36, it indicates a high fitness (the medium and low fitness ranges are 0.25 to 0.36 and 0.1 to 0.25, respectively). The goodness of fit of the model is 0.383 in this study, according to the measurements and calculations, and this indicates that the goodness of fit of the model is very high [65]. Standardized root mean square residuals (SRMRs) are also a standard for model fitting. When the SRMR value is 0, it indicates that it is perfect. However, it is recommended that an SRMR value less than 0.08 is taken as a suitable fitting threshold for PLS-SEM [60]. The SRMR value of the model in this study is 0.069, which meets the threshold value. The goodness of fit of the model is suitable according to the two tests.

Finally, collinearity problems are tested, and the VIFs between all the variables are lower than 5. Therefore, this signifies that there are no collinearity problems in this study.

### 5.4. Structural Model Results

We consider the overall explanatory power, R^2^, and path coefficient of the structural model to test the research model.

**Figure 4:**
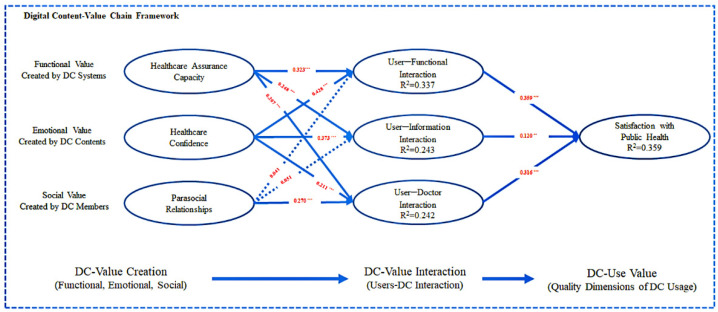
Test results of the structural model test. Note: *** *p* < 0.001 and ** *p* < 0.05.

As shown in Figure 4 and Table 6, healthcare assurance capacity has a significant positive effect on user–function interaction (ß = 0.323, *p* < 0.001), user–information interaction (ß = 0.248, *p* < 0.001), and user–doctor interaction (ß = 0.207, *p* < 0.001) impacts; thus, H1a, H1b, and H1c are supported.

Healthcare confidence has a significant positive effect on user–function interactions (ß = 0.428, *p* < 0.001), user–information interactions (ß = 0.373, *p* < 0.001), and user–doctor interactions (ß = 0.211, *p* < 0.001); thus, H2a, H2b, and H2c are supported.

Parasocial relationships have no significant positive effects on user–function interactions (ß = 0.041, *p* > 0.05) and user–information interactions (ß = 0.051, *p* > 0.05); thus, H3a and H3b are not supported. Parasocial relationships have a significant positive impact on user–doctor interactions (ß = 0.270, *p* < 0.001); thus, H3c is supported.

User–function interactions have a significant positive impact on satisfaction with public health; thus, H4a is supported. User–information interactions have a significant positive impact on satisfaction with public health (ß = 0.120, *p* < 0.05); thus, H4b is supported. User–doctor interactions have a significant positive impact on satisfaction with public health (ß = 0.316, *p* < 0.001); thus, H4c is supported.

### 5.5. Mediation Effect Results

The interactions among the user, content, and system links values are created by DC with useful values. The analysis of the value flow of DC must analyze the flow process from the value created by DC to DC use [22]. Therefore, it is necessary to perform an additional analysis to test whether the interaction between users, content, and the system has a mediation effect during the flow of a value created by mhealth apps to use value.

This study analyzes the mediating role of the model using SmartPls 3.0. As shown in Table 7, the interactions between users and doctors, information, and features in mhealth apps mediates the interactions between the healthcare coverage created by mhealth apps and users’ satisfaction with public health governance. The interactions between users and doctors, information, and features in mhealth apps mediate between the confidence in health created by mhealth apps and users’ satisfaction with public health governance. Interactions between users and doctors in mhealth apps mediate between both the value of parasocial relationships created by mhealth apps and users’ satisfaction with public health governance. However, the interactions between users and information and features in mhealth apps do not mediate between the value of the parasocial relationship created by mhealth apps and users’ satisfaction with public health governance.

## 6. Discussion and Implications

### 6.1. Discussion of Key Findings

The healthcare assurance value created by mhealth apps had a positive impact on user–function interactions, user–information interactions, and user–doctor interactions in this study, which verifies that the functional value of the DC proposed by Kim and Kim [22] promotes and expands user–system interactions. This means that the healthcare assurance value created by mhealth apps for users during the COVID-19 pandemic also actively promotes the interactions between users and information and doctors in mhealth apps. This is because mhealth apps are complicated and comprehensive health management apps under the current circumstances. Moreover, it is included in several services, such as health monitoring, appointment registration, online diagnosis, drug purchase, health knowledge search, and so on [18,19,20]. To better complete healthcare assurance, each function interacts with the users. Especially in the case of insufficient medical resources and limited travel during the COVID-19 pandemic, it needs to be completed, from health monitoring (user–function interactions) to online diagnosis (user–doctor interactions), self-health management (user–information interactions) through mhealth apps. It also confirms that the healthcare assurance value created by mhealth apps enables users to improve their satisfaction with public health governance under the intermediary role of user–function interactions, user–information interactions, and user–doctor interactions in this study. This also shows that users apply mhealth apps (DC–value interactions) to convert the DC creation value obtained from mhealth apps to their satisfaction with public health governance during the COVID-19 pandemic. It also promotes the interactions between mhealth apps and users and contributes to mhealth apps playing a greater role in healthcare assurance in public health governance during the COVID-19 pandemic.

Healthcare confidence value has a positive impact on user–function interactions, user–information interactions, and user–doctor interactions. The results prove [18,19,20] the emotional value of DC to facilitate user–content interactions and enlarge the relationships in the study. This refers to the value of mhealth apps in giving users confidence in health management during the COVID-19 pandemic while actively promoting the functions of users and mhealth apps and the interactions between doctors. Confidence improves the quality of interactions between users and mhealth apps and promotes user participation [33]. Users are confident in managing their health by using mhealth apps during the COVID-19 pandemic, so they will try their best to interact with mhealth apps. It also confirms that, under the mediation effect of these interactions, the confidence value created by mhealth apps enables users to improve their satisfaction with public health governance in the study. The results show that users convert healthcare confidence gained from mhealth apps to their satisfaction with public health governance during the COVID-19 pandemic (DC use values). Improving the various interactive experiences between mhealth apps and users will help mhealth apps play a greater role in public health governance during the COVID-19 pandemic.

In this study, the parasocial relationship values created by mhealth apps and the user–doctor interaction relationship are significant. This result verified that the social value of DC promotes user–user interactions in the Kim and Kim [22] study. This signified that the parasocial relationship can be conducive to reduce the inherent prejudice of users and improve the emotional attachment for doctors [46,47]. With the help of the parasocial relationship, users have a positive impression of doctors and tend to be satisfied with the doctor’s treatment. Finally, it can facilitate interactions between doctors and users. This study demonstrated that, under the mediation effect of user–doctor interactions, the parasocial relationship created by mhealth apps improves users’ satisfaction with public health governance. This means that users will convert the parasocial relationship attained by mhealth apps into satisfaction with public health governance during the COVID-19 pandemic. Improving the interactive experience between users and mhealth apps will help mhealth apps play a greater social role in improving doctor–patient relationships in public health governance during the COVID-19 pandemic.

However, the impact of the parasocial relationship values created by mhealth apps on user–function interactions and user–information interactions has not been verified in this study. User–function interactions and user–information interactions have no mediating role between the parasocial relationship value and public health governess satisfaction. A possible explanation is that the parasocial relationship will lead to the emotional attachment of users. After the parasocial relationship between the user and the doctor is established, the user is more likely to rely on the doctor, and he or she is unwilling to use the monitoring and information management functions in mhealth apps when he or she has health problems.

### 6.2. Theoretical Contribution

Previous studies have pointed out various functional values of mhealth apps for public health governance during the COVID-19 pandemic [16,18,19,20]. However, this study not only verified the mhealth apps’ functional value from an empirical perspective but also demonstrated the emotional and societal values for public health governance during the COVID-19 pandemic, and it clarified that, after the functional, emotional, and social values are created in mhealth apps through user–function interactions and user–information interactions, user–doctor interactions flow until satisfaction with public health governance. This provides a new view for further research on the effect of mhealth on public health governance.

In the original DC value chain framework, it confirms the relationships of functional values for user–system interactions, emotional values for user–content interactions, and social values for user–user interactions. These values flow to the value that users have used in a single way [22]. Through an empirical study of value creation via mhealth apps, this study confirmed that, in the DC value chain, the value created by DC is related to various interactions of users, and the value created can flow into the post-use value through different forms of interactions. In addition, this study further refines the functional, emotional, and social values in the DC value chain framework in specific contexts, thus expanding the dimensions of value creation in DC and enriching the antecedents that influence the interactions between users and DC. The research results of this study enrich the DC value chain framework and expand the application scope of the DC value chain framework.

### 6.3. Practical Contribution

The results of this study can provide some useful practical suggestions for the management of public health during the COVID-19 pandemic.

First, it enriches mhealth apps and expands the scope of application to guarantee the basic medical needs of users. In COVID-19, recommendation algorithms can be cleverly used to give the most appropriate results and analyze suggestions based on the questions submitted by users and suggest and associate with related functions, such as online prescriptions, face-to-face consultation appointments, etc. This can contain the users’ basic medical needs by constant interactions, which can improve their satisfaction with public health governance during COVID-19, and it is beneficial to stabilize public health order.

Second, it is necessary to provide users with healthcare confidence during the COVID-19 pandemic. This requires the public health governance department to formulate effective epidemic prevention measures in line with public opinion and pass it onto society through the efficient information distribution function of mhealth apps. Mhealth app-operating companies need to push more information about professional hospitals, public welfare organizations, and emergency hotlines to convince users that they can still get effective help when they require it urgently, even though the epidemic has affected public health order. Mobile health apps can also be embedded with interactive games (such as psychometric tests, luck predictions, etc.) to monitor changes in users’ psychological states and allow users to share positive results with friends and family to convey positive emotions.

Third, it is necessary to establish a harmonious doctor–patient relationship in the governance of public health during the COVID-19 pandemic. We suggest that the mobile app in COVID-19 needs to establish a public relations governance department to take up as much social responsibility as possible to instantly reconcile the disputes that arise in the platform. It needs to be humane when dealing with problems, taking care of the feelings of any party as much as possible, weakening conflicts, emphasizing mutual understanding, and creating an atmosphere in the platform where patients trust doctors and doctors take care of patients. Therefore, the user will be satisfied with the doctor, so the doctors also will gain self-professional identity. In this way, a benign doctor–patient relationship cycle is formed, and the governance of public health during the COVID-19 pandemic is improved.

### 6.4. Limitations and Future Research

This study has some limitations. First, this study is conducted in a city that implemented strict epidemic prevention measures (restricted travel) during the epidemic. Such results may be unsuitable for cities that implement general epidemic prevention measures. Meanwhile, many countries have begun to implement policies that coexist with COVID-19. There are no strict epidemic prevention measures; therefore, the results of this study may be unsuitable for these countries. It is necessary to compare studies with different epidemic prevention measures in future studies. Second, PLS-SEM is effective for dealing with small samples [64], but there will inevitably be representative problems due to the small sample size. Future studies should consider other methods (such as big data analysis) to analyze the value transfer process of mhealth apps during the COVID-19 pandemic. Third, the three most important specific values are listed in the study, and some value dimensions may be ignored. Therefore, further studies are necessary. Fourth, this study did not measure the effect of the length of time using the mhealth app on the value chain transmission in the COVID-19 epidemic, and future studies are necessary to analyze the effect of time of use. Fifth, since most of the mhealth apps on the Chinese market are integrated with multiple functions, the findings of this study cannot explain the value transfer of disease-specific and function-specific mhealth in COVID-19, and further research is needed. Finally, this study refers to the constant user–mhealth interaction that may not be beneficial in some studies, especially because it has a lot to do with age [66], but these problems in the effect of the mhealth app value chain are ignored, and it is suggested for future research that multigroup analyses are based on these situations.

## Figures and Tables

**Table 1:** Demographic details of the survey respondents.

Items	Options	Frequency (Total = 316)	Percentage (%)
Gender	Male	128	40.5
	Female	188	59.5
Age	18–20	55	17.4
	21–30	84	26.6
	31–40	87	27.5
	41–50	44	13.9
	51 years or above	46	14.6
Income (Per month)	RMB 1000–2000	47	14.9
	RMB 2001–3000	80	25.3
	RMB 3001–4000	71	22.5
	RMB 4001–5000	68	21.5
	More than RMB 5000	50	15.8
Education	High School	140	44.3
	Bachelor’s Degree	154	48.7
	Master or PhD Degree	22	7
mHealth app Brand	Pingan Health App	74	23.4
	Chunyu Doctor App	70	22.2
	Dinxiang Doctor App	66	20.9
	Other	106	33.5

**Table 2:** Independent *t*-test results.

Group	N	Mean (SD)	*t*-Value	Df	*p*-Value
mhealth users	316	3.063 (0.640)	9.972	291.203	0.000
Non-mhealth users	172	2.356 (0.801)			

**Table 3:** Measurement model results.

Latent Variable	Item	Loading	Mean (SD)	Cronbach’s a	CR	AVE
HAC	HAC1	0.927	3.044 (1.136)	0.856	0.913	0.777
	HAC2	0.846				
	HAC3	0.869				
ACO	ACO1	0.922	3.300 (1.080)	0.909	0.937	0.787
	ACO2	0.818				
	ACO3	0.848				
	ACO4	0.955				
PSR	PSR1	0.857	2.726 (0.672)	0.840	0.892	0.674
	PSR2	0.777				
	PSR3	0.734				
	PSR4	0.889				
UFI	UFI1	0.832	3.258 (0.807)	0.827	0.884	0.656
	UFI2	0.791				
	UFI3	0.810				
	UFI4	0.805				
UII	UII1	0.908	3.407 (0.855)	0.885	0.921	0.747
	UII2	0.798				
	UII3	0.798				
	UII4	0.942				
UDI	UDI1	0.902	3.058 (0.769)	0.817	0.880	0.648
	UDI2	0.719				
	UDI3	0.787				
	UDI4	0.801				
SPH	SPH1	0.882	3.062 (0.640)	0.837	0.891	0.673
	SPH2	0.785				
	SPH3	0.708				
	SPH4	0.892				

Abbreviations: HAC (healthcare assurance capacity); ACO (healthcare confidence); PSR (parasocial relationships); UFI (user–function interaction); UII (user–information interaction); UDI (user–doctor interaction); SPH (satisfaction with public health).

**Table 4:** Heterotrait–monotrait ratio (HTMT) test results.

	HAC	ACO	PSR	UFI	UII	UDI	SPH
HAC							
ACO	0.12						
PSR	0.434	0.284					
UFI	0.441	0.536	0.314				
UII	0.346	0.454	0.267	0.439			
UDI	0.396	0.348	0.478	0.302	0.326		
SPH	0.334	0.44	0.344	0.559	0.392	0.517	

Abbreviations: HAC (healthcare assurance capacity); ACO (healthcare confidence); PSR (parasocial relationships); UFI (user–function interaction); UII (user–information interaction); UDI (user–doctor interaction); SPH (satisfaction with public health).

**Table 5:** Fornell–Larcker criterion test results.

	HAC	ACO	PSR	UFI	UII	UDI	SPH
HAC	0.881						
ACO	0.106	0.887					
PSR	0.377	0.265	0.821				
UFI	0.383	0.473	0.276	0.81			
UII	0.307	0.413	0.243	0.385	0.864		
UDI	0.331	0.305	0.404	0.272	0.279	0.805	
SPH	0.296	0.394	0.308	0.491	0.346	0.447	0.82

Abbreviations: HAC (healthcare assurance capacity); ACO (healthcare confidence); PSR (parasocial relationships); UFI (user–function interaction); UII (user–information interaction); UDI (user–doctor interaction); SPH (satisfaction with public health).

**Table 6:** Hypothesis testing results.

Hypotheses	ß	STDEV	*t*-Statistics	*p*-Values	Result
H1a: HAC → UFI	0.323	0.045	7.148	0.000	Support
H1b: HAC → UII	0.248	0.053	4.705	0.000	Support
H1c: HAC →UDI	0.207	0.054	3.817	0.000	Support
H2a: ACO → UFI	0.428	0.043	9.933	0.000	Support
H2b: ACO → UII	0.373	0.05	7.513	0.000	Support
H2c: ACO → UDI	0.211	0.057	3.705	0.000	Support
H3a: PSR → UFI	0.041	0.05	0.819	0.413	Reject
H3b: PSR → UII	0.051	0.055	0.931	0.352	Reject
H3c: PSR → UDI	0.270	0.056	4.814	0.000	Support
H4a: UFI → SPH	0.359	0.052	6.874	0.000	Support
H4b: UII →SPH	0.120	0.049	2.456	0.014	Support
H4c: UDI → SPH	0.316	0.052	6.051	0.000	Support

Abbreviations: HAC (healthcare assurance capacity); ACO (healthcare confidence); PSR (parasocial relationships); UFI (user–function interaction); UII (user–information interaction); UDI (user–doctor interaction); SPH (satisfaction with public health).

**Table 7:** Mediation effect results.

Path	ß	STDEV	*t*-Statistics	*p*-Values
HAC → UFI → SPH	0.116	0.024	4.749	0.000
ACO → UFI → SPH	0.154	0.026	5.877	0.000
PSR → UFI → SPH	0.015	0.018	0.812	0.417
HAC → UII → SPH	0.03	0.014	2.089	0.037
ACO → UII → SPH	0.045	0.02	2.253	0.024
PSR → UII → SPH	0.006	0.008	0.783	0.434
HAC → UDI → SPH	0.066	0.02	3.21	0.001
ACO → UDI → SPH	0.067	0.02	3.331	0.001
PSR → UDI → SPH	0.085	0.025	3.392	0.001

## Data Availability

The data presented in this study are available upon request from the corresponding author. The data are not publicly available for ethical reasons.

## Appendix A

**Factor**	**Serial Num.**	**Item**	**Reference**
Healthcare assurance capacity (HAC)	HAC1	During the COVID-19 pandemic, the health management function in the mhealth app encouraged me.	Akter et al. [61]
	HAC 2	During the COVID-19 pandemic, because of the health management function in the mhealth app, I feel safe.	
	HAC 3	During the COVID-19 pandemic, the health management function in the mhealth app can solve my health problems.	
Healthcare confidence (ACO)	ACO1	During the COVID-19 pandemic, the information provided by mhealth apps allows me to know enough about my health.	Benson et al. [67]
	ACO2	During the COVID-19 pandemic, the information provided by mhealth apps makes me feel that I can take care of my health.	
	ACO3	During the COVID-19 pandemic, the information provided by mhealth apps helped me when I needed it.	
	ACO4	During the COVID-19 pandemic, the information provided by mhealth apps helped me make decisions about health management.	
Parasocial relationships (PSR)	PSR1	When I use mhealth app during the COVID-19 pandemic, the doctor I chose to make me feel like a friend.	Sokolova and Perez [68]
	PSR2	When I use mhealth apps during the COVID-19 pandemic, my communication with the doctor is very comfortable.	Zafar et al. [69]
	PSR3	When I use mhealth apps during the COVID-19 pandemic, I can rely on the doctor to provide me with a diagnosis.	
	PSR4	When I use mhealth apps during the COVID-19 pandemic, there was a small error in the doctor’s diagnosis immediately, and I will forgive him.	
User–function interaction (UFI)	UFI1	The health management function in mhealth apps is safe and reliable.	Kim and Kim [22]
	UFI2	The health management function in mhealth apps is easy to use.	
	UFI3	The steps of using health management in mhealth apps are easy to learn.	
	UFI4	The health management function in mhealth apps meets individual needs.	
User–information interaction (UII)	UII1	The information and user interaction in mhealth apps are accurate.	Kim and Kim [22]
	UII2	Information and user interaction in mhealth apps are useful.	
	UII3	The interaction between the information and the user in mhealth apps is effective.	
	UII4	The information in mhealth apps can interact with the user quickly.	
User–doctor interaction (UDI)	UDI1	Mhealth apps improve the interaction between users and doctors.	Kim and Kim [22]
	UDI2	Mhealth apps improve communication between users and doctors.	
	UDI3	Mhealth apps allow users and doctors to interact with various types of information.	
	UDI4	Mhealth apps simplify the exchange of information between users and doctors.	
Satisfaction with public health (SPH)	SPH1	During the COVID-19 pandemic, the public medical resources available to me satisfy me.	Akter, Ambra and Ray [70]
	SPH2	During the COVID-19 pandemic, I can conveniently use public medical resources.	
	SPH3	During the COVID-19 pandemic, I am very happy that I can use public medical resources.	
	SPH4	During the COVID-19 pandemic, I can use public medical resources at any time.	
